# An Analytical Comparison of the Opinions of Physicians Working in Emergency and Trauma Surgery Departments at Tabriz and Vienna Medical Universities Regarding Family Presence during Resuscitation

**DOI:** 10.1371/journal.pone.0123765

**Published:** 2015-04-23

**Authors:** Hassan Soleimanpour, Wilhelm Behringer, Jafar Sadegh Tabrizi, Kambiz Sarahrudi, Samad E J Golzari, Stefan Hajdu, Maryam Rasouli, Mehdi Nikakhtar, Robab Mehdizadeh Esfanjani

**Affiliations:** 1 Tabriz Health Services Management Research Center, Tabriz University of Medical Sciences, Tabriz-51664, I.R., Iran; 2 Department of Emergency Medicine, Medical University of Vienna, Vienna General Hospital, Waehringer Guertel 18–20, 1090 Vienna, Austria; 3 Department of Traumatology, Medical University of Vienna, Waehringer Guertel 18–20, A-1090 Vienna, Austria; 4 Cardiovascular Research Center, Tabriz University of Medical Sciences, Tabriz-51664, I.R., Iran; 5 Students’ Research Committee, Tabriz University of Medical Sciences, Tabriz-51664, I.R., Iran; 6 Department of Emergency Medicine, Tabriz University of Medical Sciences, Tabriz-51664, I.R., Iran; 7 Neurosciences Research Center, Tabriz University of Medical Sciences, Tabriz-51664, I.R., Iran; Central South University, CHINA

## Abstract

The present study evaluated the opinions of physicians working in the emergency and trauma surgery departments of Vienna Medical University, in Austria, and Tabriz Medical University, in Iran, regarding the presence of patients’ relatives during resuscitation. In a descriptive-analytical study, the data obtained from questionnaires that had been distributed randomly to 40 specialists and residents at each of the participating universities were analyzed. The questionnaire consisted of two sections aimed at capturing the participants’ demographic data, the participants’ opinions regarding their support for the family’s presence during resuscitation, and the multiple potential factors affecting the participants’ attitudes, including health beliefs, triggers that could facilitate the procedure, self-efficacy, intellectual norms, and perceived behavioral control. The questionnaire also included a direct question (Question 16) on whether the participants approved of family presence. Each question could be answered using a Likert-type scale. The results showed that the mean scores for Question 16 were 4.31 ± 0.64 and 3.57 ± 1.31 for participants at Vienna and Tabriz universities, respectively. Moreover, physicians at Vienna University disapproved of the presence of patients’ families during resuscitation to a higher extent than did those at Tabriz University (P = 0.018). Of the studied prognostic factors affecting the perspectives of Vienna Medical University’s physicians, health beliefs (P = 0.000; B = 1.146), triggers (P = 0.000; B = 1.050), and norms (P = 0.000; B = 0.714) were found to be significant. Moreover, of the studied prognostic factors affecting the perspectives of Tabriz Medical University’s physicians, health beliefs (P = 0.000; B = 0.875), triggers (P = 0.000; B = 1.11), self-efficacy (P = 0.001; B = 0.5), and perceived behavioral control (P = 0.03; B = 0.713) were significant. Most physicians at Vienna and Tabriz Medical universities were not open towards family members’ presence during resuscitation.

## Introduction

Conventionally, throughout the resuscitation procedure, in case of in-hospital cardiac arrest, patients’ relatives are guided to a separate room in which an experienced nurse advises them of the patient’s status. Permitting patients’ relatives to witness the resuscitation has always been a controversial issue. In general, relatives are rarely asked to be present in the resuscitation room, unless they are eager to be [1–5].

Throughout the international meeting of the American College of Chest Physicians in 2000, experts from all over the world presented numerous perspectives on dealing with Family Presence during Resuscitation (FPDR). At this conference, scholars emphasized that FPDR is an ethnic and cultural issue and that the results obtained from related research are country-specific and not universally applicable [6].

Given the global challenge posed by FPDR in the field of cardiopulmonary resuscitation (CPR), we resolved to evaluate the opinions of physicians working in the emergency departments of Austrian and Iranian medical universities regarding the presence of patients’ relatives during resuscitation. Our primary hypothesis was that the opinions of physicians working in the emergency and trauma surgery departments of Tabriz and Vienna medical universities (serving as models for developing and developed countries, respectively) and, therefore, within different cultures and societies, vary with regard to approval of FPDR relatives’ witnessing of patients’ resuscitation.

## Methods

Using a descriptive-analytical approach, the data obtained from 40 questionnaires that had been distributed randomly among specialists and residents in emergency and trauma surgery departments of Imam Reza Hospital, Tabriz University of Medical Sciences, Iran and Vienna General Hospital, Vienna University of Medical Sciences, Austria were captured and analyzed. The questionnaire is shown in Table 1. Tabriz Imam Reza Hospital is a 300-bed tertiary general hospital and Vienna General Hospital is a 2500-bed tertiary hospital. Overall, 32 and 35 questionnaires were returned and analyzed in Vienna General Hospital and Tabriz Imam Reza Hospital, respectively. The response rate was 80% (32/40) for Vienna General Hospital and 87.5% (35/40) for Tabriz Imam Reza Hospital.

**Table 1:** Likert scores (in parentheses) for answers to each questionnaire item.

		Strongly agree	Agree	Indifferent	Disagree	Strongly disagree
Q1	Patients’ relatives endure grief after experiencing FPDR.	(1)	(2)	(3)	(4)	(5)
Q2	Patients’ relatives will have a better understanding of the resuscitation process.	(1)	(2)	(3)	(4)	(5)
Q3	Patients’ relatives can talk to the dying patient.	(1)	(2)	(3)	(4)	(5)
Q4	Seeing the resuscitation process is a traumatic experience for family members.	(5)	(4)	(3)	(2)	(1)
Q5	The following question should be included in our departmental checklist: Does the patient’s family want to be present during CPR or not?	(1)	(2)	(3)	(4)	(5)
Q6	Patients’ relatives have the right to be in the resuscitation room.	(1)	(2)	(3)	(4)	(5)
Q7	There are many people in our department who support FPDR.	(1)	(2)	(3)	(4)	(5)
Q8	My clinical practice is affected by the presence of a patient’s family.	(5)	(4)	(3)	(2)	(1)
Q9	My supervisor expects me to allow patients’ relatives to be present during resuscitation.	(1)	(2)	(3)	(4)	(5)
Q10	The resuscitation team’s stress levels will increase as a result of the presence of a patient’s family.	(5)	(4)	(3)	(2)	(1)
Q11	Ending the resuscitation process would be difficult in the presence of a patient’s family.	(5)	(4)	(3)	(2)	(1)
Q12	The patient’s relatives may believe that the resuscitation process was disorganized.	(5)	(4)	(3)	(2)	(1)
Q13	FPDR would increase the likelihood of litigation.	(5)	(4)	(3)	(2)	(1)
Q14	If absent, relatives would be angry at staff, due to the belief that they did not exhaust their efforts.	(5)	(4)	(3)	(2)	(1)
Q15	FPDR is a privacy breach, regardless of the patient’s prior consent.	(5)	(4)	(3)	(2)	(1)
Q16	I support the legalization of FPDR.	(1)	(2)	(3)	(4)	(5)
Q17	If I were a patient’s relative, I would like to be present during resuscitation.	(1)	(2)	(3)	(4)	(5)
Q18	If I were a patient undergoing resuscitation, I would like my relatives to be present during resuscitation.	(1)	(2)	(3)	(4)	(5)

This study was approved by the Ethics Committee of Tabriz University of Medical Sciences and the Research Committee of the Vienna Emergency Medicine Department. Prior to the completion of the questionnaire, the participants received information on FPDR through a typed letter attached to the distributed questionnaires. Written informed consent was obtained by the first author of this manuscript (HS).

FPDR was defined as the presence of a family member or relative in the CPR room environment, providing them with the opportunity to witness the CPR procedure. The questionnaire consisted of the following two sections: Section 1, which was aimed at collecting the participants’ demographic data, and Section 2, which consisted of 18 questions evaluating participants’ general opinions regarding their support for FPDR and multiple factors possibly affecting their attitudes (Table 1).

Valid behavioral theories and models that informed the questionnaire items included the Health Belief Model [7], Theory of Reasoned Action [8], programmed behavior [9], and the Theory of Self-Efficacy [10]. Since no solid theory or model has ever been used to predict behavioral patterns, we combined several behavioral theories to design the questionnaire, which was based on questionnaires that had been validated in a previous study [11]. Questions 1, 2, 3, 4, 17, and 18 are related to health beliefs (i.e., if health experts believe that FDOR is useful to the patients’ health and/or the family’s psychological health). Questions 5, 6, and 7 evaluated the triggers that could facilitate FPDR initiation. Question 8 evaluated if clinical practice is affected by the presence of a patient’s family. Question 9 focuses on the intellectual norms of the participants (i.e., experiencing pressure from a superior to allow FPDR). Questions 10–15, which evaluate perceived behavioral control, reflect the participants’ conceptualization of obstacles and risks associated with their approval of FPDR. In Question 16, the participants were directly asked as to whether they approved of FPDR or not. Each of the 18 questions was rated on a Likert scale (1 = strongly support, 2 = support, 3 = indifferent, 4 = do not support, and 5 = do not support at all) [7]. To test for consistency in the participants’ responses, some of the questions were reverse-scored. Table 1 presents the real scores assigned to all the questions. The effect of each predictor (i.e., health beliefs, triggers, self-efficacy, and intellectual norms) on participants’ attitudes towards FPDR was presented as the mean of the Likert score for each corresponding question. Linear regression was used to determine the effect of the independent variables (i.e., health beliefs, triggers, self-efficacy, and intellectual norms) on the dependent variable (approval of FPDR). The Mann-Whitney *U*-test was used to compare the attitudes of participants from Tabriz Medical University and Vienna Medical University. A P-value of less than 0.05 was considered statistically significant.

## Results

Of the 32 respondents at Vienna Medical University, 7 (21.9%) were emergency medicine specialists, 21 (65.6%) were trauma surgeons, and 4 (12.5%) were emergency medical residents. Of the 35 respondents at Tabriz Medical University, 10 (28.6%) were emergency medicine specialists and 25 (71.4%) were residents (Table 2).

**Table 2:** Participants’ demographic characteristics according to affiliation.

		Vienna	Tabriz	P-value
Sex	Male	25 (78.1%)	21 (60%)	0.11
	Female	7 (21.9%)	14 (40%)	
Experience	Percentile 25	3	2	0.005
	Percentile 50	5	3	
	Percentile 75	14	6	
Age		35.67 ± 7.76	33.88 ± 6.72	0.32

The responses to each of the 18 questions are presented in Table 3.

**Table 3:** Number (%) of participants subscribing to each response category and means obtained for each question.

		City	Strongly agree	Agree	Indifferent	Disagree	Strongly disagree	Likert score	P-value
	**Health beliefs**	Tabriz						3.45 ± 1.10	0.374
		Vienna						3.81 ± 0.44	
**Q1**	Patients’ relatives endure the grief process after experiencing FPDR.	Tabriz	2 (5.7)	1 (2.9)	6 (17.1)	16 (45.7)	10 (28.6)	3.88 ± 1.65	0.056
		Vienna	0 (0)	1 (3.1)	0 (0)	18 (56.3)	13 (40.6)	4.34 ± 0.65	
**Q2**	Patients’ relatives will have a better understanding of the resuscitation process.	Tabriz	3 (8.6)	16 (17.1)	5 (14.3)	11 (31.4)	10 (28.6)	3.54 ± 1.31	0.153
		Vienna	0 (0)	1 (3.1)	0 (0)	26 (81.3)	5 (15.6)	4.09 ± 0.53	
**Q3**	Patients’ relatives can touch or talk to the dying patient.	Tabriz	3 (8.6)	6 (17.1)	7 (20)	7 (20)	12 (34.3)	3.54 ± 1.35	0.022
		Vienna	0 (0)	0 (0)	2 (6.3)	17 (53.1)	13 (40.6)	4.34 ± 0.60	
**Q4**	Seeing the resuscitation process is a traumatic experience for family members.	Tabriz	3 (8.6)	13 (37.1)	6 (17.1)	7 (20)	6 (17.1)	3 ± 1.28	0.001
Vienna	0 (0)	1 (3.1)	1 (3.1)	30 (93.8)	0 (0)	2.09 ± 0.39	
**Q17**	If I were a patient’s relative, I would like to be present during resuscitation.	Tabriz	5 (14.3)	7 (20)	1 (2.9)	7 (20)	15 (42.9)	3.57 ± 1.55	0.547
		Vienna	1 (3.1)	1 (3.1)	0 (0)	21 (65.6)	9 (28.1)	4.12 ± 0.83	
**Q18**	If I were a patient undergoing resuscitation, I would like my relatives to be present during resuscitation.	Tabriz	7 (20)	8 (22.9)	2 (5.7)	7 (20)	11 (31.4)	3.200 ± 1.58	0.084
		Vienna	2 (6.3)	2 (6.3)	6 (18.8)	9 (28.1)	13 (40.6)	3.90 ± 1.20	
	**Triggers**	Tabriz						3.22 ± 0.78	0.0001
		Vienna						4.21 ± 0.53	
**Q5**	The following question should be included in our departmental checklist: Does the patient’s family want to be present during CPR or not?	Tabriz	3 (8.6)	1 (2.9)	8 (22.9)	13 (37.1)	10 (28.6)	3.74 ± 1.17	0.017
		Vienna	1 (3.1)	0 (0)	0 (0)	17 (53.1)	14 (43.8)	4.34 ± 0.78	
**Q6**	Patients’ relatives have the right to be present in the resuscitation room.	Tabriz	2 (5.7)	7 (20)	4 (11.4)	12 (34.3)	10 (28.6)	3.600 ± 1.26	0.079
		Vienna	0 (0)	0 (0)	1 (3.1)	23 (71.9)	8 (25)	4.21 ± 0.49	
**Q7**	There are many people in our department who support FPDR.	Tabriz	7 (20)	16 (45.9)	6 (17.1)	5 (14.3)	1 (2.9)	2.34 ± 1.05	0.0001
		Vienna	0 (0)	1 (3.1)	1 (3.1)	24 (75)	6 (18.8)	4.09 ± 0.58	
	**Self efficacy**	Tabriz						2.88 ± 1.36	0.003
		Vienna						1.93 ± 0.50	
**Q8**	My clinical practice is affected by the presence of a patient’s family.	Tabriz	4 (11.4)	10 (28.6)	7 (20)	6 (17.1)	8 (22.9)	2.88 ± 1.36	0.003
		Vienna	0 (0)	1 (3.1)	0 (0)	17 (53.1)	14 (43.8)	1.93 ± 0.50	
	**Norms**	Tabriz						3.37 ± 0.91	0.0001
		Vienna						4.40 ± 0.55	
**Q9**	My supervisor expects me to allow patients’ relatives to be present during resuscitation.	Tabriz	1 (2.9)	5 (14.3)	11 (31.4)	16 (45.7)	2 (5.7)	3.37 ± 0.9	0.0001
		Vienna	0 (0)	0 (0)	1 (3.1)	17 (53.1)	14 (43.8)	4.40 ± 0.55	
	**Perceived behavioral control**	Tabriz						2.91 ± 0.88	0.001
		Vienna						2.34 ± 0.31	
**Q10**	The emotional stress of the resuscitation team will increase as a result of the presence of a patient’s family.	Tabriz	5 (14.3)	7 (20)	4 (11.4)	11 (31.4)	8 (22.9)	2.71 ± 1.40	0.081
		Vienna	0 (0)	0 (0)	1 (3.1)	31 (96.9)	0 (0)	2.03 ± 0.17	
**Q11**	Ending the resuscitation process would be difficult in the presence of a patient’s family.	Tabriz	3 (8.6)	6 (17.1)	2 (5.7)	15 (42.9)	9 (25.7)	2.40 ± 1.28	0.649
		Vienna	0 (0)	2 (6.3)	0 (0)	28 (87)	2 (6.3)	2.06 ± 0.56	
**Q12**	The patient’s relatives may believe that the resuscitation process was disorganized.	Tabriz	1 (2.9)	9 (25.7)	5 (14.3)	14 (40)	6 (17.1)	2.57 ± 1.14	0.01
		Vienna	5 (15.6)	26 (81.3)	0 (0)	1 (3.1)	0 (0)	1.90 ± 0.53	
**Q13**	FPDR would increase the likelihood of litigation.	Tabriz	3 (8.6)	13 (37.1)	8 (22.9)	4 (11.4)	7 (20)	3.02 ± 1.29	0.001
		Vienna	0 (0)	0 (0)	1 (3,1)	27 (84.4)	4 (12.5)	1.90 ± 0.39	
**Q14**	If absent, relatives would be angry at staff, due to the belief that they did not exhaust their efforts.	Tabriz	9 (25.7)	14 (40)	7 (20)	4 (11.4)	37 (13.4)	3.74 ± 1.06	0.0001
		Vienna	13 (40.6)	13 (40.6)	3 (9.4)	3 (9.4)	0 (0)	4.12 ± 0.94	
**Q15**	FPDR is a privacy breach, regardless of the patient’s prior consent.	Tabriz	3 (8.6)	13 (37.1)	8 (22.9)	5 (14.3)	6 (17.1)	3.05 ± 1.25	0.0001
		Vienna	0 (0)	0 (0)	1 (3.1)	29 (90.6)	1 (1.3)	2.00 ± 0.25	
	**Support for FPDR**	Tabriz						3.57 ± 1.31	0.018
		Vienna						4.31 ± 0.64	
**Q16**	I support the legalization of FPDR.	Tabriz	4 (11.4)	3 (8.6)	7 (20)	11 (31.4)	10 (28.6)	3.57 ± 1.31	0.018
		Vienna	0 (0)	1 (3.1)	0 (0)	19 (59.4)	12 (37.5)	4.31 ± 0.64	
		Vienna	2 (6.3)	2 (6.3)	6 (18.8)	9 (28.1)	13 (40.6)	3.90 ± 1.20	

No significant relationship was observed between Iranian and Austrian physicians’ (represented by Tabriz and Vienna Medical universities, respectively) age and FPDR (P = 0.5 and P = 0.9, respectively). The mean Likert score obtained for Question 16 was 4.31 ± 0.64 and 3.57 ± 1.31 for the participants at Vienna and Tabriz Medical universities, respectively. Vienna physicians disapproved of FPDR more so than did Tabriz physicians; this result was significant (P = 0.018). Of the studied prognostic factors affecting the views of the Vienna physicians regarding FPDR, health beliefs (P = 0.000; B = 1.146), triggers (P = 0.000; B = 1.050), and intellectual norms (P = 0.000; B = 0.714) were found to be significant. This means that these three factors significantly influenced Vienna physicians’ aversion towards FPDR. In contrast, of the studied prognostic factors affecting the views of the Tabriz physicians regarding FPDR, health beliefs (P = 0.000; B = 0.875), triggers (P = 0.000; B = 1.11), self-efficacy (P = 0.001; B = 0.5), and perceived behavioral control (P = 0.03; B = 0.713) proved significant. This means that these three factors significantly influenced Tabriz physicians’ aversion towards FPDR. The mean Likert scores obtained for each prognostic factor by the participants from the two groups are presented in Table 3. A comparison of the two groups revealed a statistically significant difference for triggers (P = 0.0001), self-efficacy (P = 0.003), intellectual norms (P = 0.0001), and perceived behavioral control (P = 0.001).

## Discussion

The results obtained from our study showed that most physicians at Vienna and Tabriz Medical universities disapprove of/strongly disapprove of FPDR; however, the proportion is much lower among physicians at Vienna Medical University (60% for Tabriz Medical University and 96.9% for Vienna Medical University; Table 3). Despite the fact that physicians at Tabriz Medical University had a generally more positive attitude toward FPDR than did Vienna Medical University’s physicians, most still did not approve of FPDR. Health beliefs and intellectual norms were identified as the most important factors influencing disagreement regarding FPDR, followed by self-efficacy and perceived behavioral control among physicians at Tabriz Medical University. In other words, the most important factor determining their negative attitudes toward FPDR was their skepticism regarding the efficacy of FPDR, as well as their colleagues’ disagreement regarding FPDR. No significant difference was found between the physicians at the two universities regarding health beliefs; however, triggers, norms, self-efficacy, and perceived behavioral control were found to be more predictive of Vienna Medical University physicians’ negative attitudes toward FPDR than Tabriz Medical University’s physicians attitudes. A previous study by Jabre et al. suggested that FPDR does not affect a medical team’s level of emotional stress and does not result in medico-legal claims [12]. Similar to many previous studies, the results obtained from our study revealed that FPDR is associated with increased fear of litigation, particularly among Tabriz Medical University’s physicians. This could explain these physicians’ aversion to FPDR [13]. However, considering the results of Jabre et al.’s study, physicians should cast aside their fear of medico-legal claims when performing family-witnessed CPR in their daily practice. Furthermore, in Jabre et al.’s study, FPDR was had apparent positive implications for the psychological aspects of the family members [12].

Most studies tend to focus on the experience of FPDR, with very few having focused on medical staff’s views in this regard. Most have stated that clear and precise policies are required regarding FPDR, so that sufficient psychological support can be provided to family members who choose to be present during CPR [14]. In a study by Duran et al., it was suggested that medical staff are mostly in favor of FPDR, which is in contrast with the results of our study [15]. This could have been due to the fact that, in addition to attending specialists, Duran et al.’s study involved other healthcare providers, such as nurses and non-attending specialists, who held more positive attitudes regarding FPDR. In our study, however, only the attitudes of the attending specialists and residents involved in FPDR were evaluated. According to the guidelines of the American Heart Association (AHA), medical staff should seriously consider the implementation of FPDR [14]. This is in sharp contrast with our study’s findings, in which the medical staff in both sub-groups did not seem open towards FPDR. In another study with similar results to ours, most physicians and nurses did not approve of FPDR in the cases of both pediatric and adult patients, believing that it would be of no benefit to the family during the grieving process [16, 17].

Although it is widely accepted that FPDR has a positive impact on the relatives witnessing CPR, in our study, the physicians in both countries tended not to accept this fact. Physicians at Tabriz Medical University seemed less reluctant to accept FPDR than those at Vienna Medical University. Based on the results obtained (Table 3), contributing factors for Austrian physicians were as follows: (1) significant disbelief in the argument, that relatives witnessing CPR would benefit from either talking to or touching the patient; (2) the assumption that the head of the department would disapprove of FPDR; and (3) the fear that most relatives would think of CPR as a disorganized procedure. Interestingly, the deterring factors were found to be different for Iranian physicians; these included fear of legislation, invasion of the patient’s privacy, reduced performance by the CPR team, traumatization of the witnessing relatives, and the assumption that the relatives would not doubt or question if the physicians did all they could.

Limitations of the study: The number of the attending professors was more than the residents in Vienna Medical University and also the number of the residents was more than the attending professors in Tabriz Medical University; this might have affected the significant differences observed between both studied groups. Our study was conducted in only two centers and it was of a relatively small sample size. Hence, the conclusion derived from this study might be ungeneralizable to all populations.

## Conclusion

In contrast to our hypothesis, emergency medicine and trauma surgery physicians in both Vienna and Tabriz medical universities disapproved of FPDR; however, this disapproval was more significant among Vienna than Tabriz physicians. FPDR should be assessed in every country, in consideration of country-specific traditions, culture, and religion. Therefore, knowledge of the different factors contributing towards physicians’ disapproval of FPDR in Iran and Austria would facilitate the removal of the obstacles hindering the execution of FPDR in those countries and enable health policymakers to implement related, required measures.

## Supporting Information

S1 Dataset Data for each participant according to answers to each questionnaire item and subscribing to each response category and means obtained for each question. (SAV)

S2 Dataset Demographic characteristics data for each participant according to affiliation. (SAV)

S1 Questionnaire Blank copy of the questionnaire. (DOC)
