# Supplementary material for: An Analytical Comparison of the Opinions of Physicians Working in Emergency and Trauma Surgery Departments at Tabriz and Vienna Medical Universities Regarding Family Presence during Resuscitation
Source: PLoS One. 2015 Apr 23;10(4):e0123765. doi: 10.1371/journal.pone.0123765 (PMC4408057; doi:10.1371/journal.pone.0123765)
Supplement: S1 Questionnaire — (DOC) [file pone.0123765.s003.doc]

**Table 1: Questionnaire ( blank copy)**

|  |  | **Strongly agree** | **Agree** | **Indifferent** | **Disagree** | **Strongly disagree** |
| --- | --- | --- | --- | --- | --- | --- |
| Q1 | Patients’ relatives endure grief after experiencing FPDR. |  |  |  |  |  |
| Q2 | Patients’ relatives will have a better understanding of the resuscitation process. |  |  |  |  |  |
| Q3 | Patients’ relatives can talk to the dying patient. |  |  |  |  |  |
| Q4 | Seeing the resuscitation process is a traumatic experience for family members. |  |  |  |  |  |
| Q5 | The following question should be included in our departmental checklist: Does the patient’s family want to be present during CPR or not? |  |  |  |  |  |
| Q6 | Patients’ relatives have the right to be in the resuscitation room. |  |  |  |  |  |
| Q7 | There are many people in our department who support FPDR. |  |  |  |  |  |
| Q8 | My clinical practice is affected by the presence of a patient’s family. |  |  |  |  |  |
| Q9 | My supervisor expects me to allow patients’ relatives to be present during resuscitation. |  |  |  |  |  |
| Q10 | The resuscitation team’s stress levels will increase as a result of the presence of a patient’s family. |  |  |  |  |  |
| Q11 | Ending the resuscitation process would be difficult in the presence of a patient’s family. |  |  |  |  |  |
| Q12 | The patient’s relatives may believe that the resuscitation process was disorganized. |  |  |  |  |  |
| Q13 | FPDR would increase the likelihood of litigation. |  |  |  |  |  |
| Q14 | If absent, relatives would be angry at staff, due to the belief that they did not exhaust their efforts. |  |  |  |  |  |
| Q15 | FPDR is a privacy breach, regardless of the patient’s prior consent. |  |  |  |  |  |
| Q16 | I support the legalization of FPDR. |  |  |  |  |  |
| Q17 | If I were a patient’s relative, I would like to be present during resuscitation. |  |  |  |  |  |
| Q18 | If I were a patient undergoing resuscitation, I would like my relatives to be present during resuscitation. |  |  |  |  |  |
